# Anti- and Protumorigenic Effects of PPAR**γ** in Lung Cancer Progression: A Double-Edged Sword

**DOI:** 10.1155/2012/362085

**Published:** 2012-08-12

**Authors:** Howard Li, Mary C. M. Weiser-Evans, Raphael Nemenoff

**Affiliations:** ^1^Division of Pulmonary Sciences and Critical Care Medicine, Department of Medicine, University of Colorado Denver, Aurora, CO 80045, USA; ^2^Pulmonary and Critical Care Section, Department of Medicine, Denver Veterans Affairs Medical Center, Denver, CO 80220, USA; ^3^Division of Renal Diseases and Hypertension, Department of Medicine, University of Colorado Denver, Aurora, CO 80045, USA; ^4^Division of Renal Diseases and Hypertension, Department of Medicine, University of Colorado, Anschutz Medical Campus, C-281 12700 East 19th Avenue, Aurora, CO 80045, USA

## Abstract

Peroxisome proliferator-activated receptor-**γ** (PPAR**γ**) is a member of the nuclear receptor superfamily of ligand-activated transcription factors that plays an important role in the control of gene expression linked to a variety of physiological processes, including cancer. Ligands for PPAR**γ** include naturally occurring fatty acids and the thiazolidinedione class of antidiabetic drugs. Activation of PPAR**γ** in a variety of cancer cells leads to inhibition of growth, decreased invasiveness, reduced production of proinflammatory cytokines, and promotion of a more differentiated phenotype. However, systemic activation of PPAR**γ** has been reported to be protumorigenic in some *in vitro* systems and *in vivo* models. Here, we review the available data that implicate PPAR**γ** in lung carcinogenesis and highlight the challenges of targeting PPAR**γ** in lung cancer treatments.

## 1. Introduction

 Lung cancer is the most common cause of cancer-related deaths in men and women worldwide and is responsible for 1.4 million deaths annually [1]. Each year, more people die of lung cancer than breast, colon, and prostate cancers combined. Despite improvements in surgical techniques and combined therapies, lung cancer remains a disease with a dismal prognosis. Although one-year all-stage survival increased from 32% in 1973 to 41% in 1994, overall five-year survival has remained unchanged at 14%. The five-year survival rate is 53% for cases detected when the disease is still localized, but only 15% of lung cancers are diagnosed at this early stage [2]. These data underscore the need to develop new therapeutic approaches to target lung cancer progression and metastasis.

 During the past 25 years, cancer research has made great progress in defining pathways involved in the transformation of “normal” epithelial cells to cancer cells. These studies have largely focused on the identification of somatic mutations resulting in the activation of oncogenes and the inhibition of tumor suppressor pathways. However, the pathways mediating the conversion of a cancer cell to a metastatic cancer cell remain poorly understood. In addition, it has become apparent during the last decade that progression of solid tumors to metastatic disease involves not just changes in the transformed epithelia itself, but also critical changes in the surrounding stroma, designated the tumor microenvironment (TME) [3]. Changes in the TME have been observed for a long time, in particular, an association between chronic inflammation and tumor development [4]. However, the mechanistic pathways whereby stromal cells contribute to cancer progression are only now beginning to be defined. These effects include changes in tumor angiogenesis [5], alterations in immune regulation [6], and changes in fibrosis and mechanical properties of the TME [7]. Each of these changes is mediated through complex interactions that involve crosstalk between cancer cells and multiple other cell types, including vascular cells, innate and adaptive immune cells, and fibroblasts. Defining this crosstalk at the molecular level will require the development of novel, more complex *in vitro* systems along with the use of genetic animal models. 

 The development of new therapeutic agents specifically designed to target progression of advanced metastatic disease distinct from tumor initiation raises several issues regarding the role of the TME in cancer progression. Importantly, activation of a specific pathway in different cell types might have opposing effects on tumor progression. This has been elegantly demonstrated in the case of the transcription factor NF-*κ*B. Work by Karin et al. demonstrated that in the setting of hepatocellular carcinoma, activation of NF-*κ*B in hepatocytes is protective against developing cancer [8, 9], whereas activation in macrophages promotes cancer progression [10]. These studies show paradoxically that the same pathway activated in different cell types exerts distinct and sometimes opposing roles on cancer progression. Given the complex interactions between the TME and cancer cells, understanding cell type-specific effects will be crucial when the use of systemically delivered therapeutics is considered. 

 Peroxisome proliferator-activated receptor-*γ* (PPAR*γ*) is a member of the nuclear receptor superfamily of ligand-activated transcription factors [11]. Activation of this receptor has been shown to be critical in adipocyte development. Importantly, PPAR*γ* is the target of the thiazolidinedione (TZD) class of drugs including rosiglitazone and pioglitazone. These agents have been employed for treatment of diabetes and act at least in part through sensitization of adipocytes to insulin. There has been great interest in this class of agents as chemopreventive and chemotherapeutic agents in a wide variety of cancers, including lung cancer [12, 13]. A large body of literature has demonstrated that direct activation of PPAR*γ* in a variety of cancer cells leads to inhibition of growth, decreased invasiveness, reduced production of proinflammatory cytokines, and in many cases promotion of a more differentiated phenotype. Use of this agent would, therefore, be predicted to be successful as a chemopreventive agent. However, systemic activation of PPAR*γ* has been reported to be protumorigenic in some experimental settings and *in vivo* models. This paper summarizes the available data that implicate PPAR*γ* in lung carcinogenesis and highlights the challenges of targeting PPAR*γ* in lung cancer treatments. We will also focus on how activation of this pathway in stromal cells may impact tumor progression.

## 2. Mechanisms of PPAR**γ** Action

PPAR*γ* is a member of the PPAR subfamily of nuclear receptors. Two isoforms have been identified in humans, PPAR*γ*1 and PPAR*γ*2. Whereas PPAR*γ*2 is expressed primarily in adipose tissue [14], PPAR*γ*1 is expressed in a broad range of tissues as well as several cancer cell lines, including lung cancer [15]. Similar to other nuclear receptors, PPAR*γ* consists of a DNA-binding domain and a ligand-binding domain connected by a hinge region [16]. There are two activation domains: AF-1 at the amino terminal and AF-2 at the carboxyl terminal. PPAR*γ* is a ligand-activated transcription factor that functions as a heterodimer with the retinoid X receptor to bind specific PPAR response elements (PPAR-RE). The consensus PPAR-RE site consists of a direct repeat of the sequence AGGTCA separated by a single nucleotide, which is designated the DR-1 site. Ligand binding causes a conformational change that leads to the release of corepressors and the binding of coactivators, resulting in increased transcription of target genes.

 Naturally occurring substances, such as polyunsaturated fatty acids and eicosanoids, are thought to serve as endogenous PPAR*γ* ligands. In particular, 15-deoxy-Δ12,14-prostaglandin J2 (15d-PGJ2) has been shown to activate PPAR*γ* specifically with micromolar affinity [17]. The lipoxygenase products of linoleic acid 9- and 13-HODE also have micromolar affinities for PPAR*γ* [18]. However, it is unclear whether these agents are regulators of PPAR*γ* *in vivo*, and studies have shown that endogenous levels of 15d-PGJ2 fail to change during adipocyte differentiation [19]. Synthetic activators of PPAR*γ* include the thiazolidinedione class of antidiabetic agents, such as troglitazone, rosiglitazone, and pioglitazone [20]. These compounds have effects on insulin-sensitivity and adipogenesis, which are mediated at least in part through PPAR*γ* activation. Nonsteroidal anti-inflammatory drugs also activate PPAR*γ*, albeit at concentrations higher than those required for cyclooxygenase inhibition [21].

Although TZDs directly activate PPAR*γ*, several reports suggest that stimulation of “off-target” pathways impacts their therapeutic effects. For example, Han and Roman showed that rosiglitazone inhibits Akt phosphorylation through PPAR*γ*-dependent induction of PTEN expression, but induction of AMPK phosphorylation and subsequent inhibition of p70S6 K phosphorylation by rosiglitazone occur through PPAR*γ*-independent signals [22]. Even with overexpression of dominant-negative PPAR*γ*, pioglitazone and rosiglitazone suppressed PGE_2_ in human non-small cell lung cancer (NSCLC) A549 cells, suggesting a PPAR*γ*-independent effect of TZDs. Similarly, tumor necrosis factor-related apoptosis-inducing ligand (TRAIL)-induced apoptosis by TZDs was shown to be mediated through PPAR*γ*-independent induction of death receptor-5 and downregulation of c-FLIP in NSCLC cell lines [23]. Early growth response-1 transcription factor was shown to be induced by troglitazone but not by other PPAR*γ* ligands, suggesting the proapoptotic effects of troglitazone may be independent of PPAR*γ*. Moreover, PPAR*γ* can directly bind to other transcription factors, including NF-*κ*B and Sp1 [24] leading to repression of these pathways. Therefore, the ability to engage or otherwise control regulatory elements distinct from classic PPAR response element sites complicates the spectrum of genes that may be controlled by PPAR*γ* [25] and poses an important barrier to understanding the biological role of PPAR*γ* in lung cancer.

## 3. Clinical Associations of PPAR**γ** with Lung Cancer

Preclinical studies using PPAR*γ* agonists, specifically TZDs such as rosiglitazone and pioglitazone, have been shown to inhibit tumor growth in many types of cancer. TZDs inhibit the growth of colon cancer cell lines *in vitro* [26–28] and in xenograft models [29] as well as growth of breast cancer [30–32] and prostate cancer cells [33–35]. In lung cancer, decreased expression of PPAR*γ* was correlated with poor prognosis in samples from human lung tumors [36]. Genetic variants in the PPAR*γ* gene have also been identified that are associated with a decreased risk for lung cancer [37]. Thus, PPAR*γ* expression may serve as a prognostic marker in lung cancer and polymorphisms in the PPAR*γ* gene may be a way to identify patients with increased risk for lung cancer. More recently, a retrospective study out of the Veterans Affairs (VA) system of nearly 88,000 individuals demonstrated a 33% reduction in lung cancer risk among TZD users compared with nonusers [38]. The risk reduction for colorectal and prostate cancers did not reach statistical significance, suggesting the beneficial effects of TZD use may be specific for lung cancer. Collectively, these data suggest that the PPAR*γ* pathway is a potential target for treatment of lung cancer. Indeed, as discussed below, several chemoprevention trials have been initiated that incorporate TZDs. Importantly, however, information regarding the effects of TZDs on lung cancer progression and metastasis is lacking. In fact, in the retrospective VA study discussed above, patients who had an established diagnosis of cancer were excluded from the study.

Several clinical trials have been initiated that incorporate TZDs for prevention of head and neck cancer or lung cancer. One phase II trial studying the effectiveness of pioglitazone in preventing head and neck cancer in individuals with oral leukoplakia showed that 71% of individuals treated with pioglitazone had complete or partial response, 10% had stable disease, and 19% had progressive disease (ClinicalTrials.gov NCT00099021). A major limitation of this study was early termination leading to small numbers of participants analyzed (21 total). However, these promising results have lead to a large collaborative trial that is currently recruiting participants looking at the effects of pioglitazone on oral premalignant lesions and the risk of head and neck cancer (ClinicalTrials.gov NCT00951379). Similarly, a clinical trial evaluating the chemopreventive ability of pioglitazone in subjects at risk for lung cancer is currently recruiting participants (ClinicalTrials.gov NCT00780234). However, an ongoing concern regarding these trials is the association of chronic TZD treatment with increased adverse cardiac events [39] and risk of bladder cancer [40, 41]. 

## 4. Effects of PPAR**γ** in Lung Cancer Cells

### 4.1. Antitumorigenic Effects of PPAR*γ*

Based on histological characteristics, lung cancer is classified as either small-cell lung cancer (SCLC) or non-small cell lung cancer (NSCLC), with NSCLC accounting for nearly 85% of all lung cancer cases. PPAR*γ* is expressed in both SCLC and NSCLC [42]. Several studies have demonstrated that activation of PPAR*γ* inhibits growth of multiple human NSCLC cell lines. For example, molecular overexpression of PPAR*γ* in human NSCLC cell lines inhibited anchorage-independent growth and invasiveness, promoted differentiation, and increased E-cadherin expression (a marker for sensitivity to tyrosine kinase inhibition) [43]. These changes were associated with cyclooxygenase 2 (COX-2) inhibition and reduced NF-*κ*B activity, resulting in decreased production of cytokines, such as IL-6, IL-8, and vascular endothelial growth factor (VEGF). The effects of pharmacological activation of PPAR*γ* using TZDs on human NSCLC lines have also been examined in several studies. Similar to molecular overexpression, TZD activation of PPAR*γ* promotes a more highly differentiated phenotype in multiple human NSCLC lines [44]. In addition, treatment of human NSCLC lines with PPAR*γ* ligands resulted in growth arrest, loss of capacity for anchorage-independent growth, and decreased activity and expression of matrix metalloproteinase (MMP) 2 [44], as well as apoptosis induction [23, 45, 46].

 COX-2 is an enzyme involved in the synthesis of prostaglandins that has been linked to the development of cancer (reviewed in [47]). Our laboratory has shown that activation of PPAR*γ* in NSCLC inhibited expression of COX-2 protein at the level of transcription [48]. In this study, suppression of COX-2 was mediated through increased PTEN activity, leading to decreased levels of phospho-Akt and inhibition of NF-*κ*B activity. One of the major metabolites of COX-2, prostaglandin E_2_ (PGE_2_), signals through G protein-coupled receptors designated EP receptors, which have also been implicated in the pathogenesis of NSCLC [49, 50]. PGE_2_ has also been shown to stimulate NSCLC proliferation via EP2 receptors [51]. In the study by Han and Roman, PPAR*γ* ligands inhibited human NSCLC growth by decreasing the expression of EP2 receptors through Erk signaling and both PPAR*γ*-dependent and -independent pathways. More recently, the TZDs pioglitazone and rosiglitazone have been shown to inhibit PGE_2_ production in NSCLC cells via a COX-2 independent pathway by upregulation of 15-hydroxyprostaglandin dehydrogenase [52]. Our laboratory has also shown that the TZD rosiglitazone specifically decreased expression of Snail [53], which is a transcription factor that regulates epithelial-mesenchymal transition. Suppression of Snail using short hairpin RNA silencing mimicked the effects of PPAR*γ* activation by inhibiting anchorage-independent growth, promoting acinar formation in three-dimensional culture, and inhibiting invasiveness. Suppression of Snail was also associated with the increased expression of E-cadherin and decreased expression of COX-2 and MMPs.

Recently, preclinical studies have demonstrated an antitumorigenic role of PPAR*γ*. Treatment of SCID mice bearing human NSCLC A549 tumors with the TZDs troglitazone or pioglitazone inhibited primary tumor growth and significantly inhibited the number of spontaneous lung metastatic lesions [54]. In addition to affecting the biology of the tumor cells themselves, activation of PPAR*γ* also reduced production of the tumor cell-derived cytokines CXCL8, CXCL5, and CXCL1, which are critical for angiogenesis and tumor-stromal interactions [55]. Work from our laboratory showed that in nude rats, orthotopic implantation of human NSCLC H2122 cells that overexpressed PPAR*γ* inhibited tumor growth and metastasis, and prolonged survival compared to implantation of control H2122 cells [43]. In addition, we have shown that transgenic mice overexpressing PPAR*γ* in lung distal epithelium are protected against developing tumors in a chemical carcinogenic model [48]. Collectively, these studies suggest that selective activation of PPAR*γ* in NSCLC cells is protective against lung cancer initiation, progression, and metastasis.

### 4.2. Protumorigenic Effects of PPAR*γ*

Although emerging data suggest that PPAR*γ* and PPAR*γ* ligands exert antitumorigenic effects on cancer cells, there is evidence that activation of PPAR*γ* may also have deleterious, protumorigenic effects. In contrast to lung cancer, a survey of various tumors revealed that PPAR*γ* is generally overexpressed in liposarcoma, colon, breast, and prostate carcinomas [56, 57]. In mouse models of colon cancer, activation of PPAR*γ* by the TZD troglitazone increased the frequency and size of colon tumors in both C57BL/6J-APC^Min^/+ mice [58, 59] and wild-type C57BL/6J mice [60]. Similar to lung cancer cell lines, breast cancer cell lines undergo growth arrest and differentiation when treated with synthetic PPAR*γ* ligands [30, 61]. However, transgenic mice expressing constitutively active PPAR*γ* in the mammary gland developed tumors at an accelerated rate compared to wild-type controls [62]. Interestingly, tumors in the PPAR*γ* overexpressing mice were more differentiated despite the more rapid rate of tumorigenesis. In high-grade hepatocellular carcinoma cell lines, treatment with PPAR*γ* antagonists has been shown to inhibit cell growth, colony formation, migration, and invasion [63]. Inhibition of PPAR*γ* activity has also been shown to suppress pancreatic cancer cell motility [64]. More recently, PPAR*γ* protein expression has been linked with the aggressiveness of thyroid cancer cells [65]. PPAR*γ* levels are elevated in cells derived from undifferentiated (anaplastic) thyroid cancer. Depletion of PPAR*γ* in anaplastic thyroid cancer cells resulted in decreased cell growth and invasiveness *in vitro. *Moreover, PPAR*γ*-depleted cells grew more slowly *in vivo* in flank and orthotopic thyroid tumors. Conversely, when PPAR*γ* was overexpressed in more differentiated thyroid cancer cells, there was increased growth and invasiveness *in vitro*.

**Figure 1:**
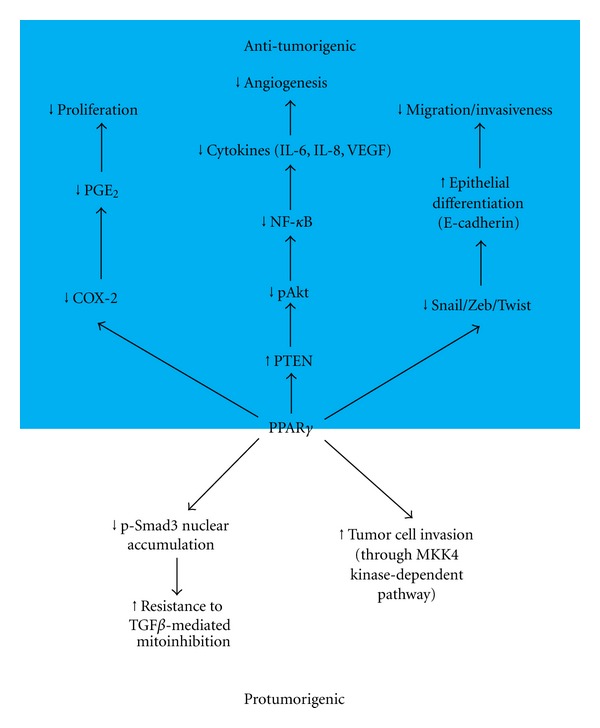
Effector pathways for PPAR*γ* in NSCLC. *Antitumorigenic effects of PPARγ on NSCLC cells (top half, shaded):* PPAR*γ*-mediated suppression of COX-2 expression in NSCLC leads to decreased PGE_2_ production, which inhibits NSCLC proliferation. PPAR*γ* can also increase expression and enzymatic activity of PTEN. This leads to inhibition of Akt activation (pAkt), and subsequent decreased activity of the transcription factor NF-*κ*B. NF-*κ*B is a transcription factor that is critical for the production of proangiogenic and proinflammatory cytokines, such as IL-6, IL-8 and VEGF. Decreased production of these factors would be expected to block tumor angiogenesis. PPAR*γ*-mediated suppression of members of the Snail family of transcription factors, such as Snail, Zeb, or Twist, would lead to derepression of E-cadherin expression and promote the epithelial phenotype, leading to decreased migration and invasiveness*. Protumorigenic effects of PPARγ on NSCLC cells (bottom half):* TGF*β*-induced PPAR*γ* has been shown to bind to Smad3 and p-Smad3, which decreases nuclear accumulation of p-Smad3 and leads to TGF*β* resistance of H460 NSCLC cells. MKK4 depletion in lung cancer cells leads to increased expression of PPAR*γ* and activation of a PPAR*γ*-dependent transcriptional program. Depletion of PPAR*γ* by shRNA in MKK4-depleted lung cancer cells has been shown to reduce invasion *in vitro. *

 In the human NSCLC line H460, which exhibits multidrug resistance, PPAR*γ* binding to both Smad3 and p-Smad3 disrupted p-Smad3-mediated mitotic arrest and growth inhibition, eventually leading to transforming growth factor-beta (TGF*β*) resistance [66]. More recently, Ahn et al. [67] demonstrated that repression of PPAR*γ*2 by mitogen-activated protein kinase kinase-4 suppressed lung cancer cell invasion. In this study, knockdown of PPAR*γ* with shRNA or treatment with the PPAR*γ* antagonist T0070907 blocked murine lung cancer cell invasion. Conversely, forced expression of PPAR*γ* enhanced murine lung cancer cell invasion. Collectively, these studies suggest that increased PPAR*γ* signaling can also serve as a tumor promoter in lung cancer. Thus, activation of PPAR*γ* in lung and other cancers can lead to either tumor suppressive or promoting responses, based on the set of conditions encountered (see Figure 1). 

## 5. Effects of PPAR**γ** Activation in the Tumor Microenvironment

**Figure 2:**
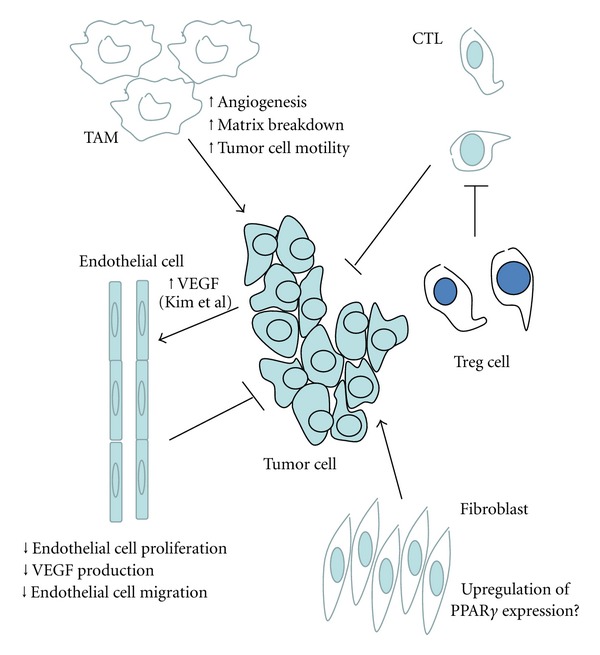
The role of PPAR*γ* signaling in the tumor microenvironment. Activation of PPAR*γ* in macrophages promotes a tumor-associated phenotype, which leads to increased tumor angiogenesis, matrix breakdown, and tumor cell motility. Activation of PPAR*γ* in myeloid cells promotes lung cancer progression and metastasis in mice. Similarly, activation of PPAR*γ* in the tumor microenvironment leads to generation of Tregs and inhibition of host T-cell antitumor activity, resulting in an immunosuppressive environment that promotes tumor progression. TZDs have been shown to inhibit angiogenesis by decreasing endothelial cell proliferation and migration, inducing endothelial cell apoptosis, and by decreasing VEGF production. However, activation of PPAR*γ* by 15d-PGJ2 has been shown to upregulate VEGF expression in human breast cancer cells, which may contribute to increased tumor angiogenesis. Finally, PPAR*γ* expression has been shown to be upregulated in stromal myofibroblasts surrounding colon adenocarcinomas, which promote proliferation, mobility, and invasion of tumor cells.

The role of the TME in mediating tumor progression has become evident over the past few years [3]. In contrast to cancer initiation, which is largely mediated through alterations in transformed epithelial cells, tumor progression and metastasis involves critical interactions between the tumor and the microenvironment. Interactions between tumor cells, vascular cells, fibroblasts, and immune cells establish a local microenvironment that suppresses the immune response and promotes cancer progression. Cancer progression involves numerous changes, including tumor angiogenesis and the acquisition of a more aggressive cancer cell phenotype. In addition to epithelial cells, PPAR*γ* is expressed in immune cells, endothelial cells and fibroblasts in and surrounding lung tumors [42]. However, the role of PPAR*γ* in the tumor microenvironment on cancer progression has not been well studied. Here, we will focus on how activation of PPAR*γ* in different stromal cells may impact tumor progression (see Figure 2).

### 5.1. Tumor-Associated Macrophages

Macrophages play a complex role in cancer progression [68–70]. Although macrophages can mediate direct cytotoxic effects on tumors, tumor-associated macrophages (TAMs) have been implicated in the promotion of tumor growth and metastasis. Specifically, TAMs can produce epidermal growth factor, which stimulates migration of tumor cells [71]. TAMs produce many proteases, including cathepsins, matrix metalloproteinases (MMPs), and serine proteases [72]. These proteases destroy the matrix to allow the escape of tumor cells from the confines of the basement membrane and migration of tumor cells through the dense stroma. TAMs are also major contributors to the angiogenic switch, which is a dramatic enhancement of vascular density that accelerates the transition to malignancy [73]. TAM-mediated angiogenesis occurs through increased accumulation of VEGF in the TME, either through production of VEGF [74, 75] or activation of MMP9, which releases VEGF from extracellular depots [76]. Thus, TAMs play a significant role in vascular remodeling as tumors progress to late carcinoma stages [77]. In addition, TAMs have been shown to affect the adaptive immune system. Production of IL-10 by TAMs inhibits cytotoxic T cell responses, resulting in expression of programmed death ligand (PD)-L1 and CCL22 and regulation of regulatory T cell influx. TAMs also suppress immune responses through synthesis of PGE_2_ and TGF*β* [78]. Finally, TAMs play a critical role in metastasis by aiding tumor cell extravasation and promoting tumor cell survival in the circulation, thereby enhancing metastatic cell seeding efficiency [79].

 Macrophages have been shown to have different activation states. “Classically” activated macrophages are educated by IFN-*γ* and LPS and are characterized by an IL-12^high^, IL-10^low^ phenotype [80]. In general, classically activated macrophages defend the host from viral and microbial infections, fight against tumors, produce high amounts of inflammatory cytokines, and activate the immune response. In contrast, “alternatively” activated macrophages are educated by IL-4 and IL-13 and are characterized by an IL-12^low^, IL-10^high^ phenotype. Alternatively activated macrophages promote scavenging of debris, angiogenesis, and remodeling and repair of wounded or damaged tissues. Importantly, alternatively activated macrophages attenuate the inflammatory response by downregulating innate immunity. Changes in macrophage phenotype have been reported during the initiation and progression of chemically induced lung tumors [81] with TAMs exhibiting an alternatively activated phenotype [82]. 

Interestingly, systemic administration of TZDs has been shown to be protective against the progression of atherosclerosis. PPAR*γ* activation in human atherosclerotic lesions primes human monocytes into alternatively activated macrophages [83, 84] thus enhancing the anti-inflammatory properties of these macrophages leading to plaque stabilization. PPAR*γ* controls the inflammatory response of macrophages by interfering with proinflammatory signaling pathways such as AP-1, NF-*κ*B, and STAT-3 [85]. Consistent with these effects, targeted deletion of PPAR*γ* in macrophages has been shown to increase atherosclerosis [86]. Thus, the antiatherogenic effects of PPAR*γ* are mediated at least in part by alternative activation of macrophages, which leads to resolution of inflammation. However, similar to macrophages residing in PPAR*γ*-activated atherosclerotic lesions, TAMs also exhibit an alternatively activated, anti-inflammatory phenotype [68, 82]. Alternative activation of macrophages in the setting of cancer progression may therefore promote tumor progression by facilitating angiogenesis, matrix breakdown, and tumor cell motility. Indeed, data from our laboratory indicate that macrophage-specific PPAR*γ* plays a critical role in the ability of cancer cells to educate macrophages into an alternatively activated phenotype [87]. Whereas selective activation of PPAR*γ* in human NSCLC cells leads to fewer metastases and increased survival in nude athymic rats [43], systemic activation of PPAR*γ* in both cancer cells and the tumor microenvironment by pioglitazone leads to increased tumor progression and metastasis in an orthotopic mouse model of lung cancer. Moreover, targeted deletion of PPAR*γ* in myeloid cells using loxP recombination promoted significantly fewer metastases in our orthotopic model [87]. We believe these findings indicate PPAR*γ* in the tumor microenvironment, and in particular TAMs, plays a critical role in lung cancer metastasis.

### 5.2. T Lymphocytes

Regulatory T cells (Tregs) found in lung tumors have been shown to inhibit the host immune response and contribute to the progression of cancer. Elimination of CD4^+^CD25^+^ Tregs elicited immune responses to syngeneic tumors in mice, leading to the eradication of the tumors [88]. Human lung tumors have been shown to contain large numbers of CD4^+^CD25^+^ Tregs, which have constitutively high-level expression of CD152 (CTLA-4). These Tregs mediated potent inhibition of autologous T cell proliferation but failed to inhibit the proliferation of allogeneic T cells [89]. Tumors formed by the CT26 colon carcinoma-derived cell line in BALB/c mice facilitated the induction or recruitment of CD4^+^ Tregs that secreted IL-10 and TGF*β* and suppressed effector CD8^+^ T cell responses [90]. Thus, Tregs could be responsible for inhibiting host T-cell activity against tumor-associated antigens.

 IL-2 is a T cell growth factor that augments NK cell cytolytic activity, contributes to the development of Tregs, and regulates the proliferation and apoptosis of activated T cells. The PPAR*γ* ligands troglitazone and 15d-PGJ2 inhibit IL-2 production in human peripheral blood T-cells in a dose-dependent manner [91]. Similarly, 15d-PGJ2 and ciglitazone inhibit proliferation and IL-2 secretion in murine helper T cells. PPAR*γ* activation also increases retinoic acid secretion from murine splenic dendritic cells, leading to induction of Tregs in the periphery [92]. Thus, activation of PPAR*γ* in the tumor microenvironment appears to lead to generation of Tregs and inhibition of host T-cell antitumor activity, resulting in an immunosuppressive environment that promotes tumor progression.

### 5.3. Tumor Angiogenesis

Tumor angiogenesis is crucial in the early stages of tumor development by allowing tumors to establish a blood supply, and in later stages of tumor progression by promoting hematogenous spread of cancer cells and metastasis. Cancer cells and bone marrow-derived myeloid cells have been shown to contribute to tumor angiogenesis through their production of growth factors, cytokines, and matrix metalloproteinases (reviewed in [93, 94]). PPAR*γ* has been shown to be highly expressed in tumor endothelium and is activated by rosiglitazone in cultured endothelial cells [95]. Panigrahy and colleagues demonstrated that rosiglitazone had both direct and indirect antiangiogenic effects by inhibiting endothelial cell proliferation and decreasing VEGF production. A more recent study has shown that pioglitazone and rosiglitazone inhibit bFGF- and VEGF-induced angiogenesis in a chick chorioallantoic membrane model [96]. In this study, endothelial cell migration was also inhibited by both pioglitazone and rosiglitazone. The PPAR*γ* ligand 15d-PGJ2 has also been shown to induce endothelial cell apoptosis [97], suggesting the PPAR pathway may be a therapeutic target for tumor angiogenesis. However, activation of PPAR*γ* by 15d-PGJ2 upregulates VEGF expression in human breast cancer cells via induction of heme oxygenase-1 and phosphorylation of ERK1/2 [98], which may contribute to increased angiogenesis of the tumor cells. 

### 5.4. Cancer Associated Fibroblasts

Myofibroblasts are unique smooth muscle-like fibroblasts that occupy a pivotal role in the stromal changes associated with carcinogenesis [99]. In response to cancer cell-derived cytokines such as TGF*β*, fibroblasts differentiate into myofibroblasts. These myofibroblasts in turn secrete proinvasive signals such as cytokines, chemokines, growth factors, and extracellular matrix proteins and proteases that promote proliferation, mobility, and invasion of adjacent epithelial cells. Expression of COX-2 in myofibroblasts indicates that these cells may also be responsible for secretion of prostaglandins such as PGE_2_, which promotes tumor invasiveness and angiogenesis [100]. PPAR*γ* expression has been shown to be upregulated in stromal myofibroblasts surrounding colon adenocarcinomas [101]. Although PPAR*γ* ligands have been shown to inhibit TGF*β*-stimulated profibrotic differentiation of lung fibroblasts *in vitro* and to reduce lung scarring in animal models of pulmonary fibrosis [102], the role of myofibroblast-derived PPAR*γ* in cancer progression remains unknown.

### 5.5. Hepatic Stellate Cells

Because the liver is a common site of metastases for many cancers, including lung, it has been hypothesized that the liver provides a prometastatic microenvironment for cancer cells, and that hepatic stellate cells (HSCs) are the predominant cell type involved with establishment of this microenvironment (reviewed in [103]). In response to paracrine factors released by cancer cells, HSCs transdifferentiate into myofibroblasts that can promote tumor growth. Activated HSCs produced growth factors and cytokines which enhance the proliferation and migration of tumor cells [104]. HSCs also promoted tumor angiogenesis by producing factors such as VEGF and angiopoietin [105–108] and have been shown to inhibit T cell proliferation and induce T cell apoptosis [109, 110], which suggests they may suppress the antitumor immune response in the liver. PPAR*γ* is expressed in quiescent HSCs, and its expression and activity decrease in HSC activation both *in vitro* and *in vivo* [111–113]. PPAR*γ* agonists inhibited HSC proliferation and chemotaxis, and expression of monocyte chemotactic protein-1 at the gene and protein levels in HSCs [111]. Similarly, forced expression of PPAR*γ* reversed culture-activated HSCs to a quiescent phenotype [114, 115]. Thus, maintenance of the quiescent state of HSC appears to require PPAR*γ*, and depletion of PPAR*γ* may be required for activation of HSCs. Although the role of HSC-specific PPAR*γ* in liver metastasis has not been established, it is likely that PPAR*γ* signaling is involved in the formation of a prometastatic microenvironment.

### 5.6. Other Immune Cells in the Tumor Microenvironment

 The tumor microenvironment is comprised of a variety of inflammatory cells, including dendritic cells, natural killer (NK) cells, myeloid-derived suppressor cells, neutrophils, and eosinophils. A recent study suggests that dendritic cells initiate antitumoral T cell responses and are pivotal for the establishment of an *in situ *efficient immune reaction in NSCLC [116]. Of note, PPAR*γ* has been shown to modulate the inflammatory response of human dendritic cells, with ligand-induced activation of PPAR*γ* by rosiglitazone resulting in enhanced phagocytosis of apoptotic neutrophils [117]. NK cells have also been implicated in the immune defense against tumors [118]. The PPAR*γ* ligands 15d-PGJ2 and ciglitazone have been shown to reduce IFN-*γ* production and inhibit cytolytic activity of human NK cells [119]. The role of PPAR*γ* in other cell types in the tumor microenvironment, however, is largely unknown.

## 6. “On-Target” versus “Off-Target” Effects of TZDs

A critical issue in interpreting studies of PPAR*γ* that utilize TZDs and other PPAR*γ* agonists is determining whether the effects of these agents are mediated through PPAR*γ*-dependent versus PPAR*γ*-independent pathways. One approach is to compare the responses of cells to TZDs with overexpression of full-length PPAR*γ*. For example, overexpression of PPAR*γ* in NSCLC cells had no significant effects on cell proliferation, as seen with TZD treatment, but instead had selective effects on anchorage-independent growth and invasiveness [48]. Specific PPAR*γ* antagonists can also be used to identify PPAR*γ*-specific effects of TZDs. Han and Roman showed that the specific PPAR*γ* antagonist GW9662 failed to affect rosiglitazone-mediated phosphorylation of AMP-activated protein kinase *α* in NSCLC cells, which indicate these effects of rosiglitazone are PPAR*γ*-independent [22]. Transfection of small interfering RNA (siRNA) or small hairpin RNA (shRNA) to silence PPAR*γ* can also help define the role of PPAR*γ* in responses to TZDs. For example, Yen and coworkers reduced PPAR*γ* levels in tumor cells using siRNA, which abolished rexinoid-mediated inhibition of invasion [120]. These data indicated that the inhibitory effects of the rexinoid bexarotene on tumor cell invasion were dependent on PPAR*γ* activation.

Defining the off-target effects of TZDs such as rosiglitazone and pioglitazone will be critical in developing new therapeutic agents. In particular, it will be important to determine whether adverse effects of these agents (e.g., increased cardiovascular events or increased incidence of bladder cancer) are mediated through PPAR*γ*-dependent or -independent mechanisms. Newer generation PPAR*γ* activators may provide more selective engagement of PPAR*γ*-dependent antitumorigenic pathways while minimizing adverse PPAR*γ*-independent cardiovascular or protumorigenic effects.

## 7. Conclusions and Implications for Therapy

The studies reviewed above implicate PPAR*γ* in lung cancer cell biology. Many studies indicate that activation of PPAR*γ* in cancer cells leads to differentiation and induction of apoptosis, which has resulted in considerable excitement regarding the use of TZDs and PPAR*γ* agonists for the prevention and treatment of lung cancer. Tumor-promoting effects of PPAR*γ* and PPAR*γ* agonists need further investigation, and the effects of PPAR*γ* activation on lung cancer cells may vary depending on tumor type or stage. In many of the studies reviewed above, it is unclear whether the biological responses of PPAR*γ* agonists are mediated through “on-target” activation of PPAR*γ*, or through other “off-target” effects. A strategy to address this issue is the use of molecular approaches, either by overexpressing or silencing PPAR*γ* in cancer cells to complement studies with pharmacological agents. Genetic mouse models using targeted knockouts of PPAR*γ* in either cancer cells or cells in the tumor microenvironment will also be informative. There will also be concerns regarding the safety of TZDs, especially since rosiglitazone use has been associated with an elevated risk of heart attacks [121], and pioglitazone use may be associated with an increased risk of bladder cancer [40, 41]. Thus, defining the molecular targets of TZDs that mediate specific responses in lung cancer cells will be critical for the development of future therapeutic interventions. Finally, the role of PPAR*γ* in cells in the tumor microenvironment remains unclear. Indeed, activation of PPAR*γ* in macrophages, Tregs, and NK cells may lead to an immunosuppressive environment that promotes tumor progression. Thus, as our laboratory has demonstrated, activation of PPAR*γ* in both tumor cells and in cells in the tumor microenvironment by systemic agents will likely have opposing effects on tumor progression. Agents that selectively activate PPAR*γ* in epithelial and cancer cells would therefore be very attractive for the prevention and treatment of lung cancer.

## References

[1] (2007). *Global Cancer Facts & Figures 2007*.

[2] SEER Cancer Statistics Review, 1975–2008. http://seer.cancer.gov/csr/1975_2008/.

[3] Kenny PA, Lee GY, Bissell MJ (2007). Targeting the tumor microenvironment. *Frontiers in Bioscience*.

[4] Grivennikov SI, Greten FR, Karin M (2010). Immunity, Inflammation, and Cancer. *Cell*.

[5] Weis SM, Cheresh DA (2011). Tumor angiogenesis: molecular pathways and therapeutic targets. *Nature Medicine*.

[6] Mlecnik B, Bindea G, Pagès F, Galon J (2011). Tumor immunosurveillance in human cancers. *Cancer and Metastasis Reviews*.

[7] Shieh AC (2011). Biomechanical forces shape the tumor microenvironment. *Annals of Biomedical Engineering*.

[8] Maeda S, Kamata H, Luo JL, Leffert H, Karin M (2005). IKK*β* couples hepatocyte death to cytokine-driven compensatory proliferation that promotes chemical hepatocarcinogenesis. *Cell*.

[9] Sakurai T, He G, Matsuzawa A (2008). Hepatocyte necrosis induced by oxidative stress and IL-1*α* release mediate carcinogen-induced compensatory proliferation and liver tumorigenesis. *Cancer Cell*.

[10] Naugler WE, Sakurai T, Kim S (2007). Gender disparity in liver cancer due to sex differences in MyD88-dependent IL-6 production. *Science*.

[11] Tontonoz P, Spiegelman BM (2008). Fat and beyond: the diverse biology of PPAR*γ*. *Annual Review of Biochemistry*.

[12] Keshamouni VG, Han S, Roman J (2007). Peroxisome proliferator-activated receptors in lung cancer. *PPAR Research*.

[13] Nemenoff RA, Weiser-Evans M, Winn RA (2008). Activation and molecular targets of peroxisome proliferator-activated receptor-*γ* ligands in lung cancer. *PPAR Research*.

[14] Fajas L, Auboeuf D, Raspé E (1997). The organization, promoter analysis, and expression of the human PPARgamma gene. *The Journal of Biological Chemistry*.

[15] Allred CD, Kilgore MW (2005). Selective activation of PPAR*γ* in breast, colon, and lung cancer cell lines. *Molecular and Cellular Endocrinology*.

[16] Laudet V (1997). Evolution of the nuclear receptor superfamily: early diversification from an ancestral orphan receptor. *Journal of Molecular Endocrinology*.

[17] Kliewer SA, Lenhard JM, Willson TM, Patel I, Morris DC, Lehmann JM (1995). A prostaglandin J_2_ metabolite binds peroxisome proliferator-activated receptor *γ* and promotes adipocyte differentiation. *Cell*.

[18] Nagy L, Tontonoz P, Alvarez JGA, Chen H, Evans RM (1998). Oxidized LDL regulates macrophage gene expression through ligand activation of PPAR*γ*. *Cell*.

[19] Bell-Parikh LC, Ide T, Lawson JA, McNamara P, Reilly M, FitzGerald GA (2003). Biosynthesis of 15-deoxy-delta12, 14-PGJ_2_ and the ligation of PPARgamma. *Journal of Clinical Investigation*.

[20] Lehmann JM, Moore LB, Smith-Oliver TA, Wilkison WO, Willson TM, Kliewer SA (1995). An antidiabetic thiazolidinedione is a high affinity ligand for peroxisome proliferator-activated receptor *γ* (PPAR*γ*). *The Journal of Biological Chemistry*.

[21] Lehmann JM, Lenhard JM, Oliver BB, Ringold GM, Kliewer SA (1997). Peroxisome proliferator-activated receptors *α* and *γ* are activated by indomethacin and other non-steroidal anti-inflammatory drugs. *The Journal of Biological Chemistry*.

[22] Han S, Roman J (2006). Rosiglitazone suppresses human lung carcinoma cell growth through PPAR*γ*-dependent and PPAR*γ*-independent signal pathways. *Molecular Cancer Therapeutics*.

[23] Zou W, Liu X, Yue P, Khuri FR, Sun SY (2007). PPAR*γ* ligands enhance TRAIL-induced apoptosis through DR5 upregulation and c-FLIP downregulation in human lung cancer cells. *Cancer Biology and Therapy*.

[24] Chen F, Wang M, O'Connor JP, He M, Tripathi T, Harrison LE (2003). Phosphorylation of PPAR*γ* via active ERK1/2 leads to its physical association with p65 and inhibition of NF-*κβ*. *Journal of Cellular Biochemistry*.

[25] Argmann CA, Cock TA, Auwerx J (2005). Peroxisome proliferator-activated receptor *γ*: the more the merrier?. *European Journal of Clinical Investigation*.

[26] Kitamura S, Miyazaki Y, Hiraoka S (2001). PPAR*γ* agonists inhibit cell growth and suppress the expression of cyclin D1 and EGF-like growth factors in ras-transformed rat intestinal epithelial cells. *International Journal of Cancer*.

[27] Shao J, Sheng H, DuBois RN (2002). Peroxisome proliferator-activated receptors modulate K-Ras-mediated transformation of intestinal epithelial cells. *Cancer Research*.

[28] Kim JA, Park KS, Kim HI (2002). Troglitazone activates p21Cip/WAF1 through the ERK pathway in HCT15 human colorectal cancer cells. *Cancer Letters*.

[29] Sarraf P, Mueller E, Jones D (1998). Differentiation and reversal of malignant changes in colon cancer through PPAR*γ*. *Nature Medicine*.

[30] Mueller E, Sarraf P, Tontonoz P (1998). Terminal differentiation of human breast cancer through PPAR*γ*. *Molecular Cell*.

[31] Yin F, Wakino S, Liu Z (2001). Troglitazone inhibits growth of MCF-7 breast carcinoma cells by targeting G1 cell cycle regulators. *Biochemical and Biophysical Research Communications*.

[32] Clay CE, Namen AM, Atsumi GI (1999). Influence of J series prostaglandins on apoptosis and tumorigenesis of breast cancer cells. *Carcinogenesis*.

[33] Mueller E, Smith M, Sarraf P (2000). Effects of ligand activation of peroxisome proliferator-activated receptor *γ* in human prostate cancer. *Proceedings of the National Academy of Sciences of the United States of America*.

[34] Hisatake JI, Ikezoe T, Carey M, Holden S, Tomoyasu S, Koeffler HP (2000). Down-regulation of prostate-specific antigen expression by ligands for peroxisome proliferator-activated receptor *γ* in human prostate cancer. *Cancer Research*.

[35] Kubota T, Koshizuka K, Williamson EA (1998). Ligand for peroxisome proliferator-activated receptor *γ* (Troglitazone) has potent antitumor effect against human prostate cancer both in vitro and in vivo. *Cancer Research*.

[36] Sasaki H, Tanahashi M, Yukiue H (2002). Decreased perioxisome proliferator-activated receptor gamma gene expression was correlated with poor prognosis in patients with lung cancer. *Lung Cancer*.

[37] Chen D, Jin G, Wang Y (2008). Genetic variants in peroxisome proliferator-activated receptor-*γ* gene are associated with risk of lung cancer in a Chinese population. *Carcinogenesis*.

[38] Govindarajan R, Ratnasinghe L, Simmons DL (2007). Thiazolidinediones and the risk of lung, prostate, and colon cancer in patients with diabetes. *Journal of Clinical Oncology*.

[39] (2007). Thiazolidinediones and cardiovascular disease. *Medical Letter on Drugs and Therapeutics*.

[40] Lewis JD, Ferrara A, Peng T (2011). Risk of bladder cancer among diabetic patients treated with pioglitazone: interim report of a longitudinal cohort study. *Diabetes Care*.

[41] Piccinni C, Motola D, Marchesini G, Poluzzi E (2011). Assessing the association of pioglitazone use and bladder cancer through drug adverse event reporting. *Diabetes Care*.

[42] Inoue KI, Kawahito Y, Tsubouchi Y (2001). Expression of peroxisome proliferator-activated receptor (PPAR)-*γ* in human lung cancer. *Anticancer Research*.

[43] Bren-Mattison Y, Van Putten V, Chan D, Winn R, Geraci MW, Nemenoff RA (2005). Peroxisome proliferator-activated receptor-*γ* (PPAR*γ*) inhibits tumorigenesis by reversing the undifferentiated phenotype of metastatic non-small-cell lung cancer cells (NSCLC). *Oncogene*.

[44] Chang TH, Szabo E (2000). Induction of differentiation and apoptosis by ligands of peroxisome proliferator-activated receptor *γ* in non-small cell lung cancer. *Cancer Research*.

[45] Kim KY, Ahn JH, Cheon HG (2007). Apoptotic action of peroxisome proliferator-activated receptor-*γ* activation in human non-small-cell lung cancer is mediated via proline oxidase-induced reactive oxygen species formation. *Molecular Pharmacology*.

[46] Shankaranarayanan P, Nigam S (2003). IL-4 induces apoptosis in A549 lung adenocarcinoma cells: evidence for the pivotal role of 15-hydroxyeicosatetraenoic acid binding to activated peroxisome proliferator-activated receptor *γ* transcription factor. *Journal of Immunology*.

[47] Dannenberg AJ, Altorki NK, Boyle JO (2001). Cyclo-oxygenase 2: a pharmacological target for the prevention of cancer. *Lancet Oncology*.

[48] Bren-Mattison Y, Meyer AM, Van Putten V (2008). Antitumorigenic effects of peroxisome proliferator-activated receptor-*γ* in non-small-cell lung cancer cells are mediated by suppression of cyclooxygenase-2 via inhibition of nuclear factor-*κ*B. *Molecular Pharmacology*.

[49] Casibang M, Moody TW (2002). AH6809 antagonizes non-small cell lung cancer prostaglandin receptors. *Lung Cancer*.

[50] Yano T, Zissel G, Muller-Qernheim J, Jae Shin S, Satoh H, Ichikawa T (2002). Prostaglandin E_2_ reinforces the activation of Ras signal pathway in lung adenocarcinoma cells via EP_3_. *FEBS Letters*.

[51] Han S, Roman J (2004). Suppression of prostaglandin E_2_ receptor subtype EP_2_ by PPAR*γ* ligands inhibits human lung carcinoma cell growth. *Biochemical and Biophysical Research Communications*.

[52] Hazra S, Batra RK, Tai HH, Sharma S, Cui X, Dubinett SM (2007). Pioglitazone and rosiglitazone decrease prostaglandin E_2_ in non-small-cell lung cancer cells by up-regulating 15-hydroxyprostaglandin dehydrogenase. *Molecular Pharmacology*.

[53] Choudhary R, Li H, Winn RA, Sorenson AL, Weiser-Evans MCM, Nemenoff RA (2010). Peroxisome proliferator-activated receptor-*γ* inhibits transformed growth of non-small cell lung cancer cells through selective suppression of snail. *Neoplasia*.

[54] Keshamouni VG, Reddy RC, Arenberg DA (2004). Peroxisome proliferator-activated receptor-*γ* activation inhibits tumor progression in non-small-cell lung cancer. *Oncogene*.

[55] Keshamouni VG, Arenberg DA, Reddy RC, Newstead MJ, Anthwal S, Standiford TJ (2005). PPAR-*γ* activation inhibits angiogenesis by blocking ELR+CXC chemokine production in non-small cell lung cancer. *Neoplasia*.

[56] Tontonoz P, Singer S, Forman BM (1997). Terminal differentiation of human liposarcoma cells induced by ligands for peroxisome proliferator-activated receptor *γ* and the retinoid X receptor. *Proceedings of the National Academy of Sciences of the United States of America*.

[57] DuBois RN, Gupta R, Brockman J, Reddy BS, Krakow SL, Lazar MA (1998). The nuclear eicosanoid receptor, PPAR*γ*, is aberrantly expressed in colonic cancers. *Carcinogenesis*.

[58] Lefebvre AM, Chen I, Desreumaux P (1998). Activation of the peroxisome proliferator-activated receptor *γ* promotes the development of colon tumors in C57BL/6J-APC(Min)/+ mice. *Nature Medicine*.

[59] Saez E, Tontonoz P, Nelson MC (1998). Activators of the nuclear receptor PPAR*γ* enhance colon polyp formation. *Nature Medicine*.

[60] Yang K, Fan KH, Lamprecht SA (2005). Peroxisome proliferator-activated receptor *γ* agonist troglitazone induces colon tumors in normal C57BL/6J mice and enhances colonic carcinogenesis in *Apc*1638 N/+ *Mlh1*+/− double mutant mice. *International Journal of Cancer*.

[61] Elstner E, Müller C, Koshizuka K (1998). Ligands for peroxisome proliferator-activated receptory and retinoic acid receptor inhibit growth and induce apoptosis of human breast cancer cells in vitro and in BNX mice. *Proceedings of the National Academy of Sciences of the United States of America*.

[62] Saez E, Rosenfeld J, Livolsi A (2004). PPAR*γ* signaling exacerbates mammary gland tumor development. *Genes and Development*.

[63] Kim KR, Choi HN, Lee HJ (2007). A peroxisome proliferator-activated receptor gamma antagonist induces vimentin cleavage and inhibits invasion in high-grade hepatocellular carcinoma. *Oncology Reports*.

[64] Nakajima A, Tomimoto A, Fujita K (2008). Inhibition of peroxisome proliferator-activated receptor *γ* activity suppresses pancreatic cancer cell motility. *Cancer Science*.

[65] Wood WM, Sharma V, Bauerle KT (2011). PPAR promotes growth and invasion of thyroid cancer cells. *PPAR Research*.

[66] Lin LC, Hsu SL, Wu CL, Liu WC, Hsueh CM (2011). Peroxisome proliferator-activated receptor *γ* (PPAR*γ*) plays a critical role in the development of TGF*β* resistance of H460 cell. *Cellular Signalling*.

[67] Ahn Y-H, Yang Y, Gibbons DL (2011). Map2k4 functions as a tumor suppressor in lung adenocarcinoma and inhibits tumor cell invasion by decreasing peroxisome proliferator-activated receptor *γ*2 expression. *Molecular and Cellular Biology*.

[68] Sica A, Bronte V (2007). Altered macrophage differentiation and immune dysfunction in tumor development. *Journal of Clinical Investigation*.

[69] Condeelis J, Pollard JW (2006). Macrophages: obligate partners for tumor cell migration, invasion, and metastasis. *Cell*.

[70] Pollard JW (2004). Tumour-educated macrophages promote tumour progression and metastasis. *Nature Reviews Cancer*.

[71] Wyckoff J, Wang W, Lin EY (2004). A paracrine loop between tumor cells and macrophages is required for tumor cell migration in mammary tumors. *Cancer Research*.

[72] Egeblad M, Werb Z (2002). New functions for the matrix metalloproteinases in cancer progression. *Nature Reviews Cancer*.

[73] Zumsteg A, Christofori G (2009). Corrupt policemen: inflammatory cells promote tumor angiogenesis. *Current Opinion in Oncology*.

[74] Leek RD, Harris AL (2002). Tumor-associated macrophages in breast cancer. *Journal of Mammary Gland Biology and Neoplasia*.

[75] Lin EY, Gouon-Evans V, Nguyen AV, Pollard JW (2002). The macrophage growth factor CSF-1 in mammary gland development and tumor progression. *Journal of Mammary Gland Biology and Neoplasia*.

[76] Giraudo E, Inoue M, Hanahan D (2004). An amino-bisphosphonate targets MMP-9—expressing macrophages and angiogenesis to impair cervical carcinogenesis. *Journal of Clinical Investigation*.

[77] Lin EY, Li JF, Gnatovskiy L (2006). Macrophages regulate the angiogenic switch in a mouse model of breast cancer. *Cancer Research*.

[78] Torroella-Kouri M, Silvera R, Rodriguez D (2009). Identification of a subpopulation of macrophages in mammary tumor-bearing mice that are neither M1 nor M2 and are less differentiated. *Cancer Research*.

[79] Qian B, Deng Y, Im JH (2009). A distinct macrophage population mediates metastatic breast cancer cell extravasation, establishment and growth. *PLoS ONE*.

[80] Gordon S (2003). Alternative activation of macrophages. *Nature Reviews Immunology*.

[81] Redente EF, Orlicky DJ, Bouchard RJ, Malkinson AM (2007). Tumor signaling to the bone marrow changes the phenotype of monocytes and pulmonary macrophages during urethane-induced primary lung tumorigenesis in A/J mice. *American Journal of Pathology*.

[82] Sica A, Larghi P, Mancino A (2008). Macrophage polarization in tumour progression. *Seminars in Cancer Biology*.

[83] Odegaard JI, Ricardo-Gonzalez RR, Goforth MH (2007). Macrophage-specific PPAR*γ* controls alternative activation and improves insulin resistance. *Nature*.

[84] Bouhlel MA, Derudas B, Rigamonti E (2007). PPAR*γ* activation primes human monocytes into alternative M2 macrophages with anti-inflammatory properties. *Cell Metabolism*.

[85] Chinetti G, Fruchart JC, Staels B (2003). Peroxisome proliferator-activated receptors: new targets for the pharmacological modulation of macrophage gene expression and function. *Current Opinion in Lipidology*.

[86] Chawla A, Barak Y, Nagy L, Liao D, Tontonoz P, Evans RM (2001). PPAR-*γ* dependent and independent effects on macrophage-gene expression in lipid metabolism and inflammation. *Nature Medicine*.

[87] Li H, Sorenson AL, Poczobutt J (2011). Activation of PPAR*γ* in myeloid cells promotes lung cancer progression and metastasis. *PLoS ONE*.

[88] Shimizu J, Yamazaki S, Sakaguchi S (1999). Induction of tumor immunity by removing CD25^+^CD4^+^ T cells: a common basis between tumor immunity and autoimmunity. *Journal of Immunology*.

[89] Woo EY, Yeh H, Chu CS (2002). Cutting edge: regulatory T cells from lung cancer patients directly inhibit autologous T cell proliferation. *Journal of Immunology*.

[90] Jarnicki AG, Lysaght J, Todryk S, Mills KHG (2006). Suppression of antitumor immunity by IL-10 and TGF-*β*-producing T cells infiltrating the growing tumor: influence of tumor environment on the induction of CD4^+^ and CD8^+^ regulatory T cells. *Journal of Immunology*.

[91] Yang XY, Wang LH, Chen T (2000). Activation of human T lymphocytes is inhibited by peroxisome proliferator-activated receptor *γ* (PPAR*γ*) agonists. PPAR*γ* co-association with transcription factor NFAT. *The Journal of Biological Chemistry*.

[92] Housley WJ, O’Conor CA, Nichols F (2009). PPAR*γ* regulates retinoic acid-mediated DC induction of Tregs. *Journal of Leukocyte Biology*.

[93] Kerbel RS (2008). Tumor angiogenesis. *New England Journal of Medicine*.

[94] Murdoch C, Muthana M, Coffelt SB, Lewis CE (2008). The role of myeloid cells in the promotion of tumour angiogenesis. *Nature Reviews Cancer*.

[95] Panigrahy D, Singer S, Shen LQ (2002). PPAR*γ* ligands inhibit primary tumor growth and metastasis by inhibiting angiogenesis. *Journal of Clinical Investigation*.

[96] Aljada A, O’Connor L, Fu YY, Mousa SA (2008). PPAR*γ* ligands, rosiglitazone and pioglitazone, inhibit bFGF- and VEGF-mediated angiogenesis. *Angiogenesis*.

[97] Bishop-Bailey D, Hla T (1999). Endothelial cell apoptosis induced by the peroxisome proliferator-activated receptor (PPAR) ligand 15-deoxy-Δ12,14-prostaglandin J_2_. *The Journal of Biological Chemistry*.

[98] Kim EH, Na HK, Surh YJ (2006). Upregulation of VEGF by 15-deoxy-Δ12,14-prostaglandin J_2_ via heme oxygenase-1 and ERK1/2 signaling in MCF-7 cells. *Annals of the New York Academy of Sciences*.

[99] Powell DW, Mifflin RC, Valentich JD, Crowe SE, Saada JI, West AB (1999). Myofibroblasts. I. Paracrine cells important in health and disease. *American Journal of Physiology*.

[100] Majima M, Amano H, Hayashi I (2003). Prostanoid receptor signaling relevant to tumor growth and angiogenesis. *Trends in Pharmacological Sciences*.

[101] Vandoros GP, Konstantinopoulos PA, Sotiropoulou-Bonikou G (2006). PPAR-gamma is expressed and NF-*κ*B pathway is activated and correlates positively with COX-2 expression in stromal myofibroblasts surrounding colon adenocarcinomas. *Journal of Cancer Research and Clinical Oncology*.

[102] Sime PJ (2008). The antifibrogenic potential of PPARgamma ligands in pulmonary fibrosis. *Journal of Investigative Medicine*.

[103] Kang N, Gores GJ, Shah VH (2011). Hepatic stellate cells: partners in crime for liver metastases?. *Hepatology*.

[104] Shimizu S, Yamada N, Sawada T (2000). In vivo and in vitro interactions between human colon carcinoma cells and hepatic stellate cells. *Japanese Journal of Cancer Research*.

[105] Taura K, De Minicis S, Seki E (2008). Hepatic stellate cells secrete angiopoietin 1 that induces angiogenesis in liver fibrosis. *Gastroenterology*.

[106] Olaso E, Salado C, Egilegor E (2003). Proangiogenic role of tumor-activated hepatic stellate cells in experimental melanoma metastasis. *Hepatology*.

[107] Torimura T, Ueno T, Kin M (2004). Overexpression of angiopoietin-1 and angiopoietin-2 in hepatocellular carcinoma. *Journal of Hepatology*.

[108] Torimura T, Sata M, Ueno T (1998). Increased expression of vascular endothelial growth factor is associated with tumor progression in hepatocellular carcinoma. *Human Pathology*.

[109] Yu MC, Chen CH, Liang X (2004). Inhibition of T-cell responses by hepatic stellate cells via B7-H1-mediated T-cell apoptosis in mice. *Hepatology*.

[110] Chen CH, Kuo LM, Chang Y (2006). In vivo immune modulatory activity of hepatic stellate cells in mice. *Hepatology*.

[111] Marra F, Efsen E, Romanelli RG (2000). Ligands of peroxisome proliferator-activated receptor *γ* modulate profibrogenic and proinflammatory actions in hepatic stellate cells. *Gastroenterology*.

[112] Galli A, Crabb DW, Ceni E (2002). Antidiabetic thiazolidinediones inhibit collagen synthesis and hepatic stellate cell activation in vivo and in vitro. *Gastroenterology*.

[113] Miyahara T, Schrum L, Rippe R (2000). Peroxisome proliferator-activated receptors and hepatic stellate cell activation. *The Journal of Biological Chemistry*.

[114] Yu J, Zhang S, Chu ESH (2010). Peroxisome proliferator-activated receptors gamma reverses hepatic nutritional fibrosis in mice and suppresses activation of hepatic stellate cells in vitro. *International Journal of Biochemistry and Cell Biology*.

[115] Hazra S, Xiong S, Wang J, Rippe RA, Chatterjee VKK, Tsukamoto H (2004). Peroxisome proliferator-activated receptor *γ* induces a phenotypic switch from activated to quiescent hepatic stellate cells. *The Journal of Biological Chemistry*.

[116] Sautès-Fridman C, Cherfils-Vicini J, Damotte D (2011). Tumor microenvironment is multifaceted. *Cancer and Metastasis Reviews*.

[117] Majai G, Gogolák P, Ambrus C (2010). PPAR*γ* modulated inflammatory response of human dendritic cell subsets to engulfed apoptotic neutrophils. *Journal of Leukocyte Biology*.

[118] Waldhauer I, Steinle A (2008). NK cells and cancer immunosurveillance. *Oncogene*.

[119] Zhang X, Rodriguez-Galán MC, Subleski JJ (2004). Peroxisome proliferator-activated receptor-*γ* and its ligands attenuate biologic functions of human natural killer cells. *Blood*.

[120] Yen WC, Prudente RY, Corpuz MR, Negro-Vilar A, Lamph WW (2006). A selective retinoid X receptor agonist bexarotene (LGD1069, targretin) inhibits angiogenesis and metastasis in solid tumours. *British Journal of Cancer*.

[121] Nissen SE, Wolski K (2010). Rosiglitazone revisited: an updated meta-analysis of risk for myocardial infarction and cardiovascular mortality. *Archives of Internal Medicine*.

